# Genomic diversity analysis enables development of pan-Dengue Toehold RNA sensors

**DOI:** 10.1371/journal.pntd.0014173

**Published:** 2026-04-16

**Authors:** Anirudh Nandakumar, Deeksha Mishra, Akshay Shendre, Edrea Mendonca, Soujanya Nagendra, Akash Gulyani, Arati Ramesh

**Affiliations:** 1 National Centre for Biological Sciences, GKVK Campus, Bangalore, India; 2 Trans-Disciplinary Health Sciences and Technology, Bangalore, India; 3 Department of Biochemistry, School of Life Sciences, University of Hyderabad, Central University Post, Prof. C.R. Rao, Gachibowli, Hyderabad, Telangana, India; 4 Tata Institute for Genetics and Society, NCBS campus, GKVK Post, Bengaluru, India; 5 Academy of Scientific and Innovative Research (AcSIR), Ghaziabad, India; UCLM: Universidad de Castilla-La Mancha, SPAIN

## Abstract

The Dengue virus (DENV) like many other RNA viruses exhibits high genome sequence diversity. This poses a challenge to nucleic acid-based diagnostics, rendering them inefficient at detecting the diverse DENV strains circulating in a population. In this study, we address this challenge by developing a Toehold sensor assay that despite significant genomic diversity is able to detect ~99.4% of all strains of DENV. To this end, our custom workflow first identifies relatively conserved short stretches (36-nt) within all DENV genomes, which could potentially serve as triggers that activate Toehold RNA sensors. We then add a crucial step of mismatch-tolerance that allows related triggers with high sequence diversity and multiple mismatches in the sensor-binding region to be efficiently sensed by the same sensor. Deploying *in vitro* transcription translation assays, we show that the designed sensors were able to efficiently sense target RNA triggers from all the four DENV serotypes. These data demonstrate that multiple Toehold RNA sensors were able to tolerate limited mismatches in the target RNA sequences without compromising response. The sensitivity of the Toehold sensor assay is often increased by inclusion of an isothermal RNA amplification step. We show by employing multiple primer sets that diverse trigger RNAs from all four serotypes of the dengue virus can be successfully amplified and subsequently detected by the same sensor. Together, this approach has resulted in a pan-dengue Toehold sensor assay, which presents a powerful nucleic acid detection platform to detect viruses with high sequence diversity.

## Author summary

RNA viruses show high rates of sequence mutations, helping them evolve rapidly to evade the host immune system. This confounds diagnosis through detection tests where specific fragments from the viral genome are detected through designer sensors. The high variability in viral RNA sequences from various disease isolates makes it challenging to identify conserved regions against which sensors can be designed. Here, we report a strategy that overcomes the challenge of genomic diversity and enables design of Toehold RNA sensors that successfully detect >99.4% of all the reported strains of the dengue virus (a prominent example of a highly divergent virus). This work paves the way for RNA-diagnostics/sensing even for highly evolved RNA-viruses.

## Introduction

Over 300 different viruses are known to infect humans and cause disease. Timely detection of infection is important not only to guide treatment but also to prevent the spread of infection. One factor that continues to obscure detection is the enormous sequence diversity observed in viral genomes. The genome sequence diversity observed in viruses can be attributed to multiple factors such as the inherent rate of mutation, recombination, reassortment and antibody-mediated selection pressure [1–4]. DNA viruses have mutation rates between 10^−8^ to 10^−6^ substitutions per nucleotide site, per cell infection and this is even higher in RNA viruses which show mutation rates of 10^−6^ to 10^−4^ substitutions per nucleotide site, per cell infection [5,6].

Dengue (DENV), a positive-strand RNA virus, shows high sequence diversity, exemplified by the fact that it exists as at least four distinct serotypes 1, 2, 3, and 4 [7–9], which themselves are sub-divided into multiple genotypes. The Dengue virus causes significant morbidity, posing a major burden to healthcare systems particularly in tropical and sub-tropical countries where dengue is widely prevalent. Dengue infection may cause symptoms ranging from mild fever, severe flu like symptoms, to hemorrhagic fever including serious bleeding, drop in blood pressure and death. As per WHO estimates, there are about 100–400 million dengue infections per year, globally. Estimates suggest that as many as 3.9 billion people are at the risk of dengue infection [10,11]. This makes dengue detection a pressing challenge, requiring innovative approaches.

Diagnosis of dengue infection is often done through immunoassays against the NS1 protein [12,13], whose signature regions also help to type the virus [14]. Alternately, detection of the viral RNA through RT-qPCR assays is a recommended diagnostic test especially in the acute phase of infection [15]. Several nucleic acid detection strategies such as NASBA [16–18], RT-RPA [19–21], CRISPR-based diagnostics [22,23], and RT-LAMP [24,25] have been described, where amplification of viral RNA relies on sequence-dependent primer/guide RNA recognition. Notably, in all these methods, the sequence diversity inherent to the dengue genomes could obscure diagnosis.

To address viral sequence diversity, one approach has been to align representative DENV sequences from all genotypes/serotypes to identify the most conserved segments of a specific genomic region such as the C-prM and NS1 genes, or the 3’ UTR of the genome [25–28]. Other approaches involve exploiting the mismatch tolerance of primer-target recognition [24], or engineering mismatch tolerance into the amplification reaction by adding a proof-reading polymerase which removes 3’ end mismatches [29]. These studies along with recently reported tools for analyzing viral sequence diversity [30,31] underline the importance of considering sequence diversity while developing assays for viral detection.

A recent innovation in viral detection are the toehold RNA sensors, which have been designed to detect viruses including Zika [32,33], SARS-CoV-2 [34–38], hepatitis A [39], norovirus [40], and others reviewed here [41,42]. Toehold sensors have also been adapted to detect pathologically relevant miRNA [43] and for profiling common gut-bacteria [44]. The toehold RNA switch is a structured element placed upstream of a reporter mRNA, whose ribosome binding site (RBS) and start codon (AUG) are sequestered in a base-paired structure and hence kept inaccessible to the ribosome for translation. The sensors are designed to contain a sequence complementary to the target, that upon binding the target RNA, initiates a structural rearrangement of the sensor, enabling translation of the reporter gene. The sensors can be coupled to various reporters that enable visual (lacZ), portable (gfp, glucose oxidase), and rapid (nano-lantern) detection of the target [32,34,45,46]. When coupled with nucleic acid amplification techniques such as NASBA [32,34,40], RT-RPA [40], or RT-LAMP [35] these sensors show sensitivity comparable to RT-LAMP and RT-RPA assays. While these studies reveal that toehold RNA-based detection is versatile and meets the needs of diverse diagnostic settings, they do not comprehensively address the sequence diversity of the viral target itself. One example of viruses that are highly divergent in sequence is the norovirus. To design toehold sensors for noroviruses, a conserved region was found using sequence alignments of multiple norovirus genomes and toehold sensors were designed against the conserved region [40]. Since viruses continue to accumulate mutations over time, it becomes essential to employ a dedicated design strategy that takes into consideration this sequence divergence.

In this work, we have designed and developed a toehold RNA-based assay that overcomes DENV sequence diversity and results in a pan-dengue assay. For this, we established a computational pipeline that first identifies the 36-nucleotide regions with maximum conservation among all DENV genomes. We then exploit the inherent ability of a toehold sensor to tolerate a certain degree of mismatch with its target sequence, resulting in related triggers from very divergent DENV genomes to be sensed by the same toehold sensor. This strategy resulted in DENV- toehold sensors that could detect >99.4% of DENV sequence variations globally reported thus far, making them truly “pan-dengue” sensors. Typically, toehold sensor assays are coupled with an RNA amplification step for increased sensitivity of detection. Here, using multiple primer sets we show that diverse RNA triggers belonging to each of the four DENV serotypes could be successfully amplified and sensed by our pan-dengue sensor.

## Methods

### Estimating nucleotide level diversity in RNA viruses

Sequences of DENV (taxon lineage ID: 12637), Zika virus (ZIKV) (taxon lineage ID: 64320), Middle East Respiratory Syndrome (MERS) (taxon lineage ID: 1335626), and Norovirus (NORO) (taxon lineage ID: 142786), which were marked as complete sequences and isolated from human host, were downloaded from the BV-BRC Database [47] in FASTA [48,49], format, along with the corresponding metadata. The genomes were indexed and aligned using the Augur toolkit [50], with NC_035889.1, NC_038294.1, NC_044853.1, and NC_001474.2 as the reference genomes for ZIKV, MERS, NORO, and DENV, respectively.

The alignments were analyzed through an in-house code where only positions with occupancy greater than 50% were considered for the analysis. Shannon entropy for a given position x, was calculated using the formula: $H_{x}=-\Sigma p_{n,x}lnp_{n,x}$ where $p_{n,x}$ is the fraction of genomes that have a particular nucleotide *n* at position *x*.

### Analysis of Dengue genomes and identification of conserved regions in Dengue genomes

Genomes of DENV which were qualified as full-length and isolated from human sources were downloaded from the BV-BRC Database [47] in FASTA [48,49] format on October 20, 2021. 6,716 in total. Genomes with more than 5% low-quality positions were discarded to obtain 6,712 genomes. The genomes were grouped into their respective serotypes Dengue 1 (2595 genomes), Dengue 2 (1714 genomes), Dengue 3 (1281 genomes), and Dengue 4 (543 genomes) according to the database; sequences with no serotype assignment, were assigned to Unclassified- “UC” (579 genomes).

The multi-line genome sequence entries were appended to give a single-line genome sequence for every entry. All the genome entries along with their respective headers from each serotype were concatenated and placed under a line that started with “#” followed by the serotype specifier label.

Using a custom script, we identified all possible thirty-six nucleotide segments from all genomes in every serotype. Sequences that were composed of only the standard four nucleotides were considered. The occurrences of each trigger in all the DENV genomes was calculated. We applied a Similarity Criteria wherein we took triggers which showed 0–3 mismatches as compared to a parent trigger and pooled them together (see representative triggers in S1 Table). Each trigger in a trigger pool was analyzed for its occurrence across genomes of every serotype. This gave an estimate of the prevalence of each trigger pool across each serotype. We selected only 61 trigger pools, since they were prevalent in >96% of genomes of every serotype.

### Design of Toehold RNA sensors

Toehold sensors were designed as described earlier [34]. Briefly, toehold sensors were designed to detect the parent trigger in each trigger pool that met the criteria. The sensors were designed to detect the parent trigger since the similarity relation (0–3 mismatches) holds true only when the sequences in the trigger pool are compared to the parent trigger. This sets up the sensor with the potential to detect the variants in the trigger pool without any changes to the sensor.

**Fig 1:**
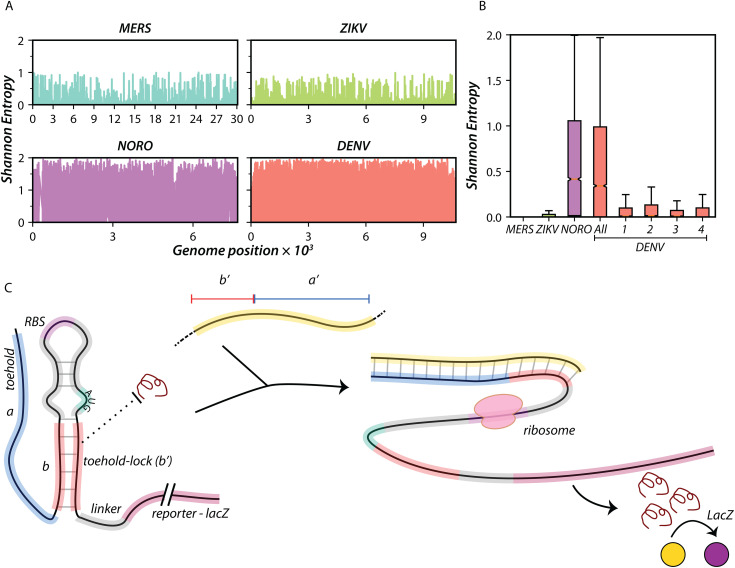
Nucleotide-level diversity in RNA viruses and concept of Toehold sensors. **(A)** Shannon entropy plots at each nucleotide position across the genomes of four ssRNA viruses are shown. MERS (tax. id: 1335626, 219 genomes), ZIKV (tax. id: 64320, 729 genomes), NORO (tax. id: 142786, 1452 genomes), and DENV (tax. id: 12637*,* 6826 genomes) are shown. Shannon entropy values = 2 indicates no conservation. **(B)** Average Shannon Entropy calculated across every nucleotide position is shown for MERS, ZIKV, NORO, DENV, and four DENV serotypes. Bars represent the mean, and error bars represent standard deviation. DENV (“All” refers to all DENV serotypes) and NORO exhibit high sequence diversity throughout the genome, compared to MERS and ZIKV. Each serotype of DENV also exhibits higher levels of diversity within them compared to MERS and ZIKV. **(C)** The toehold RNA sensor in its OFF state, is composed of a structured region (gray and red stems) juxtaposing the RBS (purple) and the start codon AUG (cyan). This structure impedes translation initiation of the downstream reporter (such as *lacZ*). The trigger RNA (yellow, segment marked *a’*) binds the toehold region (blue, segment marked a) and extends into the toehold-lock, thus unzipping the intra-molecular base-pairing *b-b’*. This allows access of the ribosome to the RBS and start codon, upregulating translation of the reporter protein.

Reverse complement of the parent trigger sequence was designed to be the toehold region of the sensor (*a* and *b* in Fig 1C) and the first 11 nucleotides of the parent trigger form the toehold-lock region (*b’* in Fig 1C). The toehold and the toehold-lock region are connected by a sequence containing the RBS and AUG, described in detail in Pardee et al., 2016 [32]. The design includes a single nucleotide position between the toehold-lock (b') region and the linker (see Fig 1C). This single nucleotide position is an A, U, G or C, resulting in four possible sensors for each parent trigger. Any toehold sensors that have a stop codon in the toehold-lock region, are discarded. After this process, we obtained 219 sensors.

We then calculated trigger single strandedness, toehold single strandedness, probability of formation of the lower stem pairing and similarity to expected sensor secondary structure. For trigger single strandedness, toehold single strandedness calculations we used the complex defect module of Nupack 3.2.2 [51]. For probability of formation of the lower stem pairing and similarity to expected sensor secondary structure we used the pairs module of Nupack 3.2.2. From the 219 sensors designed above, we obtained 67 sensors that met arbitrary criteria of Trigger SS > 0.49, Toehold SS > 0.32, lower stem score >0.97 and expected secondary structure score >0.74.

The specificity of the chosen triggers was assessed through nucleotide BLAST with the blastn algorithm against the non-redundant database excluding Dengue virus (taxid: 12637). Triggers were analyzed for their similarities (e < 1) to human or other related pathogens.

### *In vitro* transcription coupled translation assay (IVTT)

The IVTT reaction conditions were adapted from Chakravarthy et al., 2021 [34]. The assay was conducted using the NEB PURExpress kit (Cat. no. E6800L). The reaction mixture consisted of 2 μl of solution A, 1.5 μl of solution B, 0.125 μl (10 Units) of RNase Inhibitor (Thermo Fisher Scientific, Cat. no. 10777019), ChlorophenolRed-β-D-galactopyranoside-CPRG (Sigma-Aldrich, Cat. no. 59767) 0.375 μl of 12 mg/ml dye was added as a substrate, and 125 ng linear DNA template of the sensor fused to *lacZ* gene. The IVTT reactions were incubated at 37°C in 384-well plates (Corning, Cat. no. 3544) with 1.25 μl of the cognate 9 μM Trigger RNA or, 1.25 μl of NASBA products or, indicated copies of trigger RNA or, 1.25 μl of nuclease free water. The absorbance at 576 nm was monitored every 5 minutes for 2 hours in a Varioskan Lux instrument (Thermo Fisher Scientific) or Tecan Infinite M Plex, or the reactions were quenched at the end of 2 hours with 2 μl of 2 M Na_2_CO_3_ and the absorbance of the ten-fold diluted samples were recorded using the Eppendorf Biospectrometer with an Eppendorf µCuvette G1.0. The fold change in absorbance was calculated relative to the reaction where only water was added, sensor “OFF” state. In the time-course plots, data from Tecan Infinite M Plex absorbance values above 4 (above instrument saturation) were not included. For data from Varioskan Lux absorbance values above 4 have been plotted but should be looked at only for the overall trend and not for their absolute values. All absorbance-based plate reader experiments were initially baseline corrected using a blank sample, and each sample was normalized so that the lowest absorbance measurement was set to 0. Data visualization and figure creation were carried out using GraphPad Prism 8 and Adobe Illustrator, respectively.

### Preparation of trigger and template RNA

Trigger and template RNA for the IVTT and NASBA were prepared through *in vitro* transcription of corresponding DNA templates that had a T7 promoter. DNA templates of the triggers were prepared by amplifying the template oligomers with the corresponding forward and reverse primer specified in S2 Table for triggers used in Fig 3B or S3 Table for triggers used in Fig 4A–4C. The template RNA corresponding to DENV serotype 1 and 3 were constructed through a PCR that involved amplification of two long oligomers that were partially complementary, by corresponding forward and reverse primers specified in S4 Table. Template RNA corresponding to DENV serotype 2 and 4 were constructed in two steps, and the final sequence was encoded by two disjoint long oligomers. The first steps involved extending the long oligomers to incorporate elements that are complementary to the other long oligomers using primers mentioned in S4 Table. Finally, the purified products of the previous extension PCR were added in stoichiometrically equal amounts and amplified by primers specified in S4 Table.

**Fig 2:**
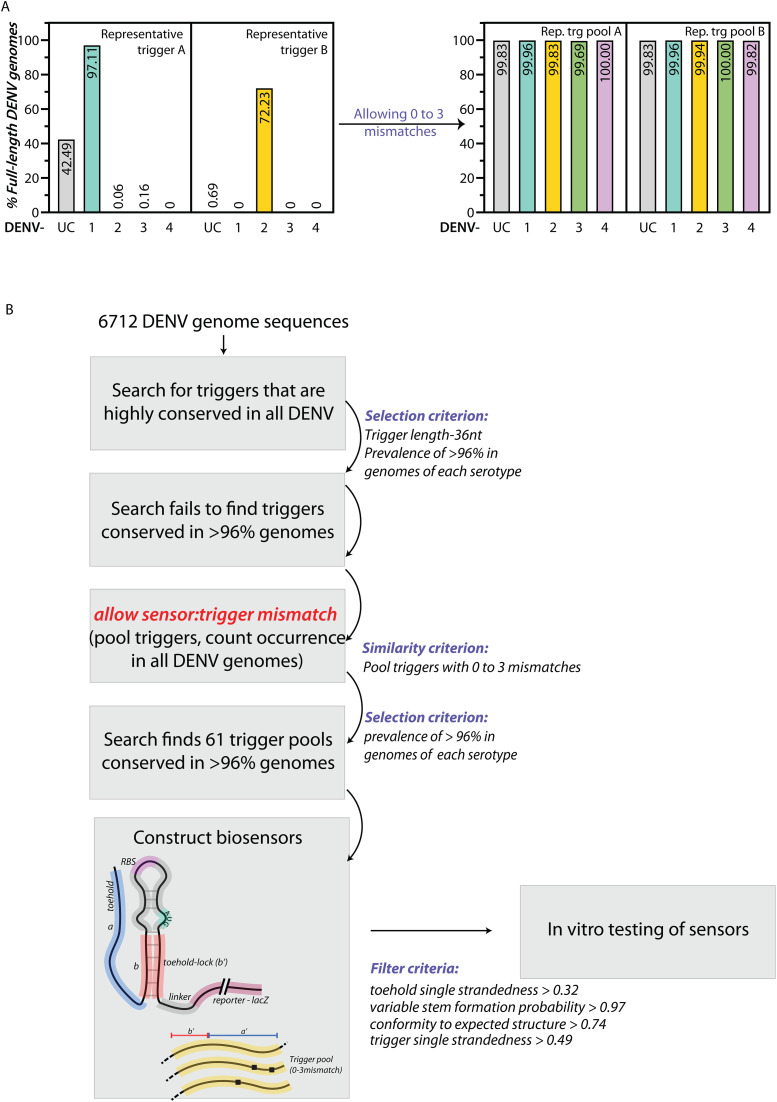
Design of Toehold RNA sensors to detect all variants of Dengue virus. **(A)** Left: Percentage of full-length DENV genomes in which a representative trigger is found, in each serotype DENV 1-4 and unclassified (UC). Pooling triggers that have 0 to 3 mismatches with Trigger A (CGTCTTTCAATATGCTGAAACGCGCGAGAAACCGCG) and B (AATATGCTGAAACGCGAGAGAAACCGCGTGTCAACT) leads to trigger pools that are present in >99.6% of DENV genomes in each serotype (Right). **(B)** Potential pan-Dengue toehold sensors were designed using a computational pipeline outlined in the flowchart. All available high-quality full-length genomes of DENV from DENV 1,2,3,4 and UC were taken. A library of unique 36-nt triggers was built and occurrence across all DENV genomes was calculated. To address low occurrence of triggers across genomes, a Similarity Criterion was applied where every trigger was pooled with triggers that varied by 0 to 3 mismatches (Hamming distance). A selection Criterion was applied where only trigger pools that were present in >96% genomes across each serotype were retained. Toehold sensors were designed to detect the parent trigger of each trigger pool. The sensors and their cognate triggers were filtered on the basis of four secondary structure parameters indicated, and taken forward for *in vitro* testing.

The RNA templates for NASBA reactions and Trigger RNAs for cell free IVTT reactions were synthesized by *in vitro* transcription reactions. This was done in a 40 μl *in vitro* transcription reaction system. Each reaction contained 1 μg of the relevant DNA template, 4 μl of 10X T7 polymerase reaction buffer (Toyobo, Cat. no. TRL-201), 5.5 μl of 50 mM MgCl_2_, 4 μl of 25 mM rNTPs (NEB, Cat. no. N0450S), 2 μl (100 units) of T7 RNA polymerase enzyme (Toyobo, Cat. no. TRL-201), 2 μl (0.2 units) Yeast Inorganic Pyrophosphatase (NEB, Cat. no. M2403S), 0.5 μl (20 units) RNaseOUT (Invitrogen, Cat. no. 10777019), and the remainder of the reaction volume was made up to 40 μl with nuclease free water and incubated at 37°C for 2 hours. After this, the samples were treated with 2 μl (4 units) of DNAse I enzyme (NEB, Cat. no. M0303S) at 37°C for 1 hour and purified using the ZymoResearch RNA Clean and Concentrator RNA purification kit (Cat. no. 1015). The final RNA sample was eluted in nuclease free water for further use.

For human RNA control, 133ng of total RNA isolated from HEK cells was added to each IVTT reaction. For COVID-19 RNA control, 133ng of “trigger 1” described in Chakravarthy et al., 2021 was added to each reaction [34].

### NASBA

The NASBA reaction performed as described previously Chakravarthy et al., 2021 [34]. The reactions were carried out by adding 10^8^ copies of template RNA with a master mix containing 4 μl of 5X AMV RT Buffer (Promega, Cat. No. M515A or Roche, Cat. no. 10109118001), 1.6 μl of 50 mM MgCl_2_, 2 μl of 25 mM rNTPs (NEB, Cat. no. N0450S), 2 μl of 10 mM dNTPs (NEB, Cat. no. N0447S), 0.18 μl of 1 M DTT (VWR, Cat. no. 3483-12-3), 3 μl of 100% DMSO (Sigma-Aldrich, Cat. no. D8418-50 Ml), 0.2 μl of 10 mg/ml BSA (Roche, Cat. no. 10735078001), and 0.5 μl each of 10 μM forward and reverse primers. The reaction mixture was made up to 17.7 μl, and the contents were heated at 65°C for 5 minutes, followed by incubation at 50°C for an additional 5 minutes. An enzyme mix containing 1 μl (50 Units) T7 RNA Polymerase (Toyobo, Cat. no. TRL-201), 1 μl (20 Units) AMV-RT (Promega, Cat. No. M510F or Roche, Cat. no. 10109118001), 0.1 μl (0.2 Units) RNaseH (Roche, Cat. no. 10786357001), and 0.2 μl (12.5 Units) of RNaseOUT (Invitrogen, Cat. no. 10777019) was added to the reaction, and incubated at 42°C for 2 hours. The reactions were stored at -80°C or used in IVTT assays. The RNA templates used in the reactions for DENV serotypes 1–4 are declared in S4 Table. The primers together covered 90% of the DENV genomes that reported at least 100 nucleotides upstream of trigger pool 3 sequences (3964 genomes). Sequences of all primers used in this study are declared in S5 Table.

## Results

### Analysis of viral genomes for sequence level diversity

We analyzed all the sequences of *Betacoronavirus cameli* (MERS), *Orthoflavivirus zikaense* (ZIKV), *Orthoflavivirus denguei* (DENV), and *Norovirus norwalkense* (NORO), to estimate the diversity in the nucleotide sequence among their genomes. Full-length genome sequences of MERS (219 genomes), ZIKV (729 genomes), DENV (6712 genomes) and NORO (1452 genomes) were taken from the Bacterial and Viral Bioinformatics Resource Center [47] database, and the Shannon entropy [52,53] for every position across the genome was calculated (Fig 1A and 1B). DENV and NORO genomes show high divergence at several genomic positions (Shannon entropy ≥ 1 in 23% and 27% of total positions respectively). In contrast, MERS and ZIKV contain no positions with Shannon entropy ≥ 1).

Dengue is caused by four different serotypes of DENV, each with a distinct serum neutralization profile. The Shannon entropy calculations reveal a high level of sequence divergence even within each serotype. While the mean Shannon Entropy for DENV and NORO are 0.539 and 0.594, and for MERS and ZIKV are 0.007 and 0.026, the mean Shannon Entropy for the individual DENV serotypes are in the range of 0.117 to 0.164. (Figs 1B and S1).

This degree of sequence divergence implies that identification of conserved genomic regions in viruses like DENV and NORO is challenging. Any pan-dengue biosensors designed to report on all dengue variants would ideally need to take into consideration this diversity. We decided to address this diversity in our design of the toehold-RNA based dengue sensors.

### Design of toehold biosensors for detection of Dengue

The toehold sensor contains a 36-nucleotide region that is complementary to a portion of the target viral RNA (Fig 1C). 11 nucleotides at the 3’ end of the 36-nt stretch are part of a base-paired structure that keeps the RBS and AUG sequestered, while the initial 25 nucleotides toehold region that engages the target (trigger) RNA is linear. Trigger-binding initiates the disruption of the structure, allowing the translation of the downstream reporter (Fig 1C).

To identify 36-nt trigger sequences that are conserved across all DENV serotypes, we took all full-length DENV genomes (6712 genomes) from the BV-BRC database and indexed all possible 36-nucleotide triggers in each genome. Occurrence of each of these triggers across every DENV genome was calculated. Even the most conserved trigger sequences were not present in every DENV serotype. For example, a representative trigger (Trigger A) appeared in 97% of genomes of a serotype and another representative trigger (Trigger B) appeared in 72% of genomes of a serotype but both these triggers were absent in other serotypes (Fig 2A left panel).

We then added a crucial step that allows mismatches between the Trigger and sensor region. This relaxation of trigger conservation was inspired by previous studies [40] which have shown that toehold sensors can also be turned on by RNA triggers that have up to 3 mismatches. Hence, we took triggers which had 0, 1, 2, or 3 sequence mismatches (within 3 Hamming distances) and pooled them together. The resulting trigger pools were selected if they were prevalent in >96% of genomes of each serotype. For example, applying the similarity criteria to the two representative triggers shown in Fig 2A results in trigger pools that appear in >99.6% of genomes of each serotype (Fig 2A right panel, S1 Table). Totally, we obtained 61 trigger pools that appear in >96% of genomes of each serotype.

Interestingly, all the 61 trigger pools map to an open reading frame encoding the capsid protein close to the start of the ORF (S2 Fig). Thus, our analysis suggests that the region within the sequence coding for the capsid protein is the least divergent in terms of sequence. The 3’ UTR of the DENV genomic RNA has been shown to make long range interactions with the 5’ of the genome and these interactions are important for replication of the DENV genome [54,55]*.* The region to which our trigger pools map, is included in this previously noted long range interaction.

**Fig 3:**
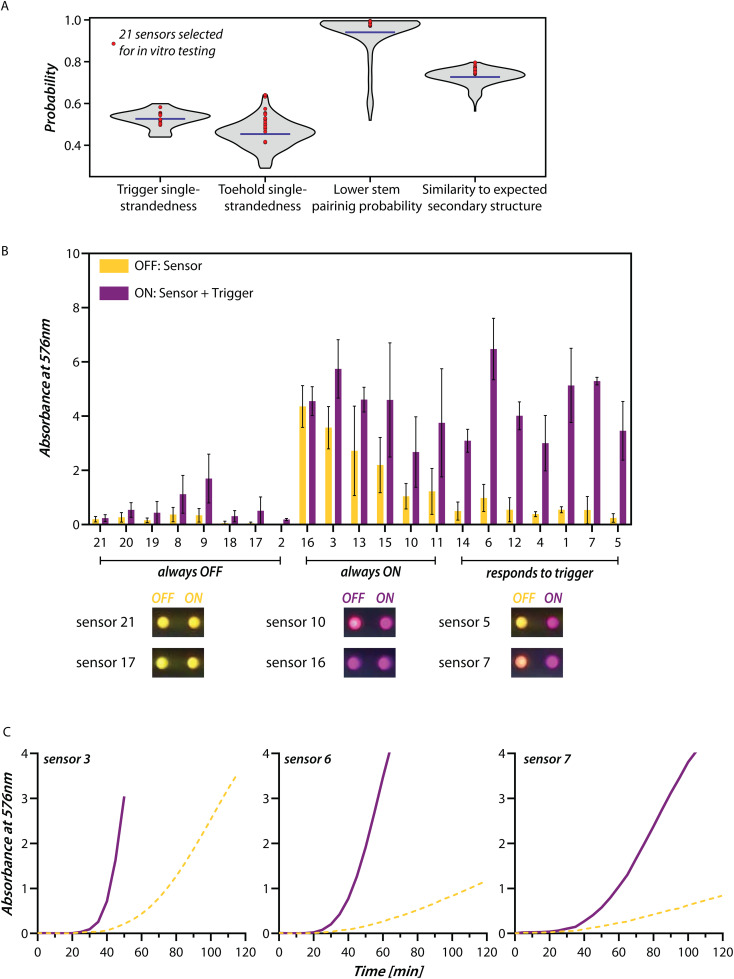
*In vitro* screening of DENV Toehold RNA sensors. **(A)** Violin plot (gray) shows the distribution of the four secondary structure parameters of 61 triggers and 219 toehold sensors. Mean value of the parameters is indicated by the blue line. Parameters of the 21 sensors chosen for *in vitro* testing are shown (Red circle). **(B)** *In vitro* transcription translation (IVTT) assay of 21 sensors in presence (purple) or absence (yellow) of their cognate triggers is shown. Absorption of CPRG dye at 576 nm was used to report the extent of translation at the end of 2h at 37°C. The sensors showed three types of responses- those that did not turn ON even in the presence of the trigger (always OFF), those that were ON even in the absence of trigger (always ON), and sensors that responded specifically to the trigger. Post-2 hour images of the IVTT reaction are shown for select sensors from the three categories mentioned above (also see S3A Fig). Bars represent mean and the error bars represent standard deviation (n = 3). **(C)** Absorbance at 576 nm was monitored over time for IVTT reactions with sensors 3, 6, and 7. Presence of trigger (purple) showed faster increase in absorbance when compared to absence of trigger (yellow). Lines represent individual time-course curves for sensor 3, 6 and 7. Additional replicates are shown in S3B Fig.

These 61 trigger pools were used to design the corresponding toehold sensors (Fig 2B and Methods). We scored the triggers and sensors on four criteria such as the single strandedness of the trigger, toehold single strandedness, probability of lower stem formation, and conformity to the designed structure of the sensor were calculated for all the designed sensors. We noted the average trigger and toehold single strandedness of the targets and sensors were 0.54 and 0.46 (Fig 3A), respectively. These values are lower than those used for selecting toehold sensors designed against SARS-CoV-2 [34] and suggest a higher degree of intra-molecular interactions in the DENV target RNAs and concomitantly in the toehold region of DENV sensors.

### Toehold sensors that report efficiently on the presence of dengue RNA

We tested 21 candidate toehold sensors coupled to the *lacZ* reporter. We measured their response to their corresponding trigger in terms of the increase in absorbance at 576 nm due to the hydrolysis of CPRG by LacZ at the end of two hours (Figs 3B and S3A). DNA corresponding to each candidate toehold sensor was used as input for an *in vitro* transcription translation (IVTT) coupled assay. *In vitro* synthesized trigger RNAs were used to check the performance of the sensors. The IVTT reactions showed three types of responses. Sensors (21, 20, 19, 8, 9, 18, 17, and 2) didn’t turn ON even in the presence of their triggers (ON < 2), sensors (16, 3, 13, 15, 10, and 11) were constitutively turned ON independent of the trigger (ON/OFF ≤ 3), and sensors (14, 6, 12, 4, 1, 7, and 5) showed a discernible change in the presence of trigger RNA compared to its absence (ON/OFF > 5). We noticed that three sensors 3, 6, and 7 showed the highest ON state values. Hence we investigated these further by performing time course experiments (Figs 3C and S3B). Interestingly, for sensor 3, while the 2-hour end-point value of the OFF state was high, it had a high ON/OFF ratio in the 40–50-minute time range with a low OFF state value. This sensor also saturates faster (A_576nm_ = 4 in 30 minutes) than sensor 6 (65 minutes) and sensor 7 (115 minutes).

### Validation of sensors for their ability to detect all Dengue serotypes

**Fig 4:**
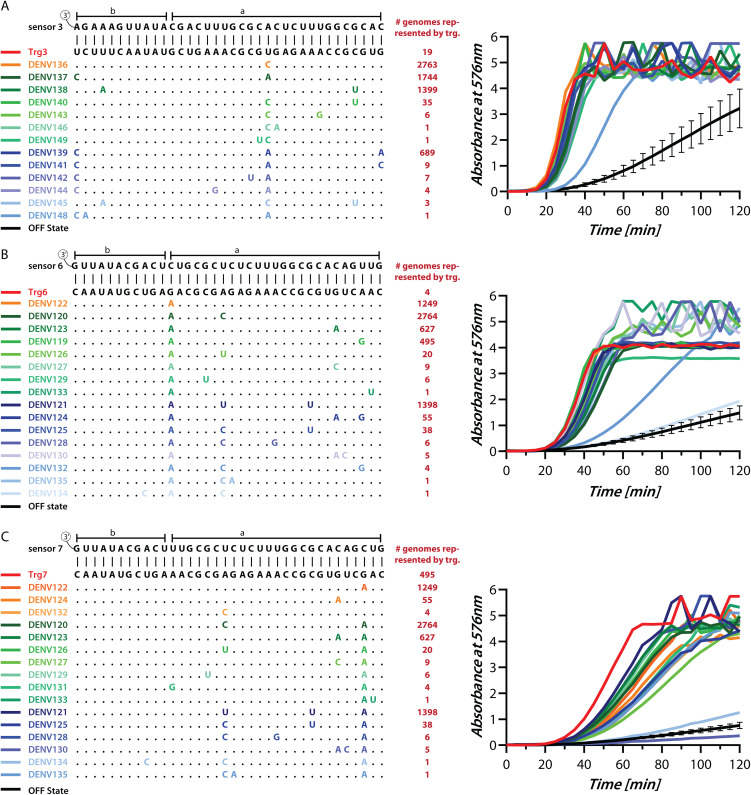
Detection of different DENV serotypes by toehold sensors. Left panel shows Multiple sequence alignment of different triggers in a trigger pool with 0 to 3 mismatches (highlighted in color) from the parent trigger (black). Also shown are the trigger binding regions of the respective sensors 3 (panel **(A)**, 6 **(B)**, and 7 **(C)**. The number of genomes that contain each trigger sequence is shown (red). Right panel shows the IVTT assay performed with a sensor in the absence (black curve is an average of all OFF states, with error bars representing standard deviation) or presence of various triggers from the trigger pool (colored lines, colors are matched with the left panel). IVTT was monitored through change in absorbance at 576 nm with 10^13^ copies of each trigger. Values above 4 have been plotted but should be looked at only for the overall trend and not for their absolute values. All three sensors detect the majority of triggers tested, accounting for >99.4% of all DENV genomes. These results show that sensors 3, 6, and 7, are pan-dengue.

We next asked if our sensors would be capable of detecting the majority of DENV genome variants. This is important given that our pan-dengue sensors could only be designed when we made an allowance of 0–3 mismatches while identifying triggers that were present in more than 96% genomes of each serotype. To test the mismatch tolerance of our sensors, we synthesized several triggers from the trigger pools for sensors 3, 6, and 7, and tested them in an IVTT assay. For sensor 3, we tested 13 different RNA triggers that represented 99.6% of all DENV genomes (Figs 4A and S4A). Sensor 3 shows a good response to all tested triggers including those with 3 mismatches between the trigger and the toehold region. Similarly, for sensor 6 (Figs 4B and S4B) and sensor 7 (Figs 4C and S4C), we tested 16 RNA triggers each, since they represented 99.6% of all DENV genomes. Sensor 6 shows a good response to 15 out of 16 tested triggers. whereas sensor 7 responds to 14 out of 16 tested triggers. Trigger DENV 130 that is not sensed by sensor 7 accounts for only 5 genomes and DENV134 that is not sensed by either sensor 6 or 7 is present only in 1 genome, hence do not represent a significant genome population. Together, these results confirm that the toehold sensors do respond to even 3 mismatches in the trigger. Importantly, sensors 3, 6 and 7 are each able to detect nearly 99.4% all DENV genomes including all known serotypes (S5A–S5C Fig). Hence these are truly pan-dengue sensors.

### Coupling of sensors with isothermal RNA amplification

A reported feature of all toehold sensors, including our pan-dengue sensors (S6 Fig) is that their limit of detection is in the order of 10^12^ to 10^13^ copies of trigger RNA [32,34,35,40,44,46]. To increase sensitivity, toehold sensor assays are typically coupled with a step of target RNA amplification like RT-LAMP [35] or NASBA [32,34,40,44]. We noted that sequence diversity in viral genomes may affect the amplification step that precedes toehold-based detection, and hence attempted to couple our pan-dengue sensor with an isothermal amplification strategy that would account for viral sequence diversity.

**Fig 5:**
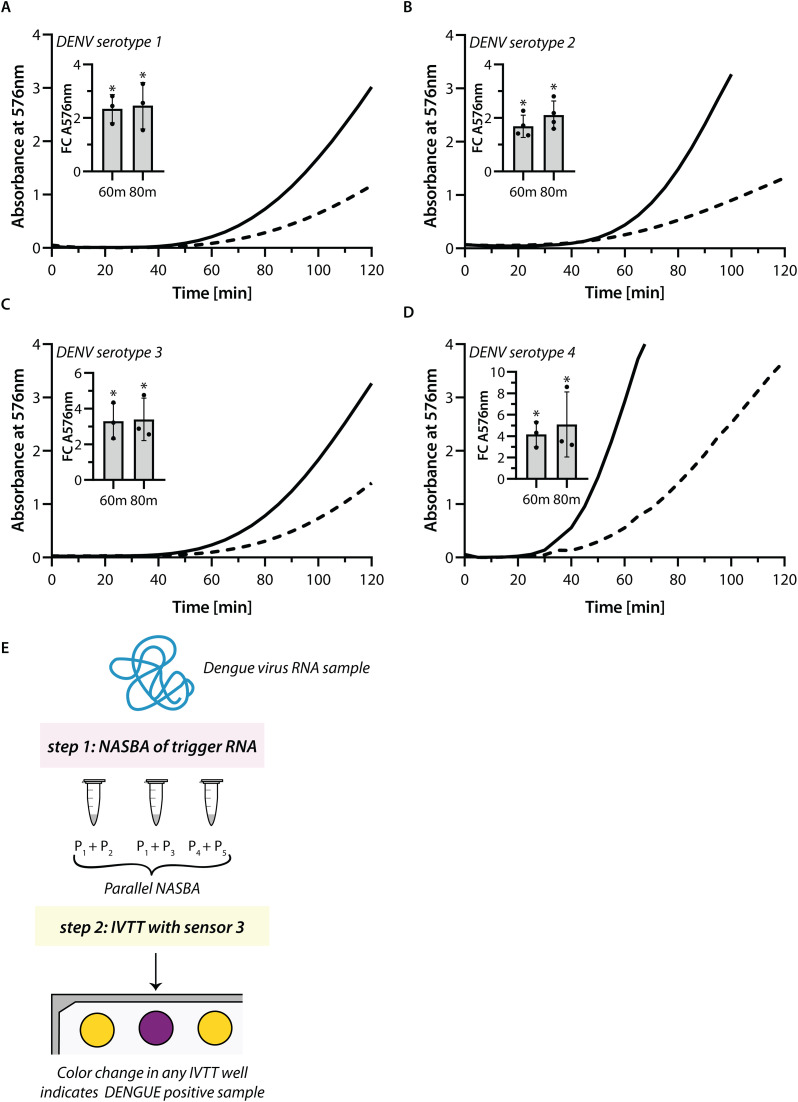
Coupling of sensors with isothermal RNA amplification. NASBA reactions were performed on templates representing different serotypes- DENV Serotype 1 **(A)**, DENV Serotype 2 **(B)**, DENV Serotype 3 **(C)**, and DENV Serotype 4 **(D)**. NASBA reactions post 2 hours were added to an IVTT reaction containing sensor 3. Change in absorbance at 576 nm was recorded over time. NASBA reactions performed on the different templates (solid lines) were compared with reactions where no template was added to the NASBA (dashed lines). Fold change in absorbance in the NASBA reactions at 60 and 80 minutes compared to the NTC is shown in inset. Inset: Bars represent mean and error bars represent standard deviation in the fold change in three or more replicates. Statistical significance was determined through unpaired t-tests. (* p < 0.05) **(E)** Schematic shows how our pan-dengue sensors may be combined with different NASBA primer sets (P1, P2, P3, P4, P5) to enable DENV diagnostics. As shown in this work, individual NASBA reactions each containing one primer pair amplifies the trigger region of a particular DENV serotype and this followed by IVTT with sensor 3 indicates presence of DENV virus in the sample.

To this end, we attempted to design NASBA primers that could amplify the regions around and including the 36-nucleotide trigger. As expected, we were unable to find any one pair of NASBA primers that could take into account the significant sequence variations within DENV genomes. Hence, we resorted to multiple primer sets that would amplify the trigger region of most genomes of a serotype, but not limited to it. To test this we first synthesized short template RNAs representative of DENV-1, 2, 3, and 4. These RNAs were used as templates in NASBA reactions with respective primer pairs (see Methods). NASBA is an isothermal amplification method where the reverse primer initiates the synthesis of the cDNA strand. RNAse H degrades the RNA from the RNA:DNA hybrid, and this is followed by second strand synthesis by a forward primer containing the T7 promoter sequence. T7 polymerase in the reaction, transcribes RNA corresponding to the region between the forward and reverse primers. This RNA serves as a template for iterative amplification of the target RNA [56]. In our experiment, NASBA was initiated with 10^8^ copies of template RNA. Upon completion, NASBA reactions were tested for their ability to turn on sensor 3 in IVTT assays. We found that sensor 3 shows a reproducible increase in absorbance in the presence of the NASBA reaction as compared to a “no-template” control where NASBA was performed without any template RNA (Figs 5A–5D and S7A–S7D). Notably, 10^8^ copies of trigger RNA by themselves were unable to elicit a response from sensor 3, but post-NASBA the target RNA was amplified to a detectable range. This indicates that our pan-dengue sensor 3 is efficiently coupled to NASBA and that the primer sets that we have designed are efficient in amplifying targets from all serotypes. As an additional control we also tested if sensor 3 shows any response to human RNA or an unrelated COVID-19 RNA. IVTT results show that human RNA (from Human Embryonic Kidney HEK cells) or the COVID-19 RNA do not turn ON the sensor whereas, the DENV trigger RNA shows clear activation of the sensor (S8 Fig).

Based on these results we propose that facile detection of DENV, a virus that shows very high sequence divergence, would be possible using toehold switches. To make this possible, NASBA using multiple primer pairs would be coupled with a pan-dengue sensor.

## Discussion

In this work, we report a strategy to develop a toehold sensor assay, for a highly evolved virus such as DENV. Our analysis of the DENV genome revealed significantly high sequence divergence even among the different serotypes of DENV. Since this posed a challenge in developing pan-dengue sensors, we came up with a strategy where we first take into account the sequence diversity of all available genomes and select targetable regions common to them. This alone was not enough and we added another step of allowing 0–3 mismatches between the target and sensor. This allowance led us to identify the most conserved and hence targetable region within DENV genomes. Based on this we designed toehold sensors, which when filtered through established toehold criteria yielded three pan-dengue sensors that we show can detect close to 99.4% of all known dengue genomes including all serotypes. We found these sensors could be coupled with NASBA, an isothermal amplification method. This results in more sensitive detection of all the DENV serotypes. Hence our sensors are truly pan-dengue and we establish a method that enables design of toehold switches for detection of highly RNA divergent viruses.

The toehold RNA switch presents a powerful method to detect viral nucleic acids since it utilizes the inter-molecular interactions between the sensor and trigger RNA, resulting in the control of translation of any reporter gene that enables color or fluorescence-based read-out. Designing toehold switches especially against RNA viruses can be challenging, since RNA viruses may exhibit very high levels of sequence divergence over time. This is ascribed to their replication mechanisms that typically lack proof-reading. DENV, NORO, HIV, and Lassa are examples of such viruses, which have evolved over time to accumulate a substantial degree of sequence variations [57–60]. While developing nucleic acid tests for such viruses, it would be vital that the design accounts for sequence divergence by taking into account all the available genome sequences. A recent report describes a toehold based sensor that responds to DENV RNA [61]. This work presented a strategy for increasing toehold sensitivity, however the reported sensor was designed only to detect a specific DENV genome, not addressing the diversity of prevalent DENV serotypes.

Crucial to our design strategy was that we allowed 0–3 mismatches between the trigger RNA and the sensor. Even with 3 mismatches we saw a clear response from all three selected sensors. This showed tremendous tolerance in the toehold’s interactions with its trigger. Triggers that were not sensed (DENV 130 by sensor 7 and DENV 134 by both sensor 6 and 7) both had 3 mismatches with their cognate sensor. DENV 134 shows low single-strandedness, coupled with an unusually high Minimal Free Energy Secondary Structure (-15.5 kcal/mol) compared to all other triggers hence it is possible that it forms stable intramolecular interactions within itself. DENV 130 bears 3 mismatches in close proximity to each other, offering a plausible reason why this is not sensed by sensor 7. A previous study designed toehold switches to distinguish between wild-type target RNA versus different Single Nucleotide Polymorphisms [62]. Here mismatches govern the opening of the sensors, further highlighting the tunability of toehold switches towards mismatches.

Our design of toehold sensors requires us to find short 36-nucleotide regions with high conservation in the genome. With this narrow criterion, we found that the most conserved region of the genome lies in the coding region of the capsid protein that along with the membrane protein and envelope protein constitute the structural proteins of DENV. This region has been shown to be involved in long range interactions with the 3’ untranslated region of the DENV genome to facilitate replication. It is possible that the importance of these interactions contributes to the high degree of conservation we see.

The motivation behind our study was to develop pan-dengue sensors that can detect almost all the sequence variations in DENV. While we achieved this with the sensors alone, we needed to couple our sensors with isothermal amplification (NASBA) for sensitive detection. This posed a problem in terms of finding conserved primers. Hence, we had to select primers from different serotypes (but not exclusive to each serotype). These primers showed sensitive detection across all 4 DENV serotypes and worked well when coupled with sensor 3. We envision a diagnostic where these primers may be used as individual primer sets in parallel reactions, followed by detection via our pan-dengue sensor. While we have demonstrated the ability of toehold RNA sensors to recognize multiple sequence variants of DENV, the efficacies of these sensors for patient samples and the true limit of detection for these DENV sensors remains to be determined.

## Supporting information

S1 Fig Extent of diversity within genomes of each DENV serotype. A) Diversity in the composition of nucleotides at each position across the genomes of DENV serotypes 1–4 is shown in terms of Shannon entropy.
(EPS)

S2 Fig All 61 pan-dengue trigger pools identified in this study are located in the Capsid protein coding region of the DENV genome (top). Sequences of the parent trigger of all 61 pan-dengue trigger pools were aligned using MAFFT [63] and have been shaded on the basis of nucleotide conservation. Darker shades indicate higher nucleotide conservation.
(EPS)

S3 Fig A. *In vitro* screening of DENV Toehold RNA sensors. *In vitro* transcription translation (IVTT) assay of 21 sensors in presence (ON) or absence (OFF) of their cognate triggers was performed. CPRG, a yellow-coloured substrate of LacZ added to the reaction, gets hydrolyzed by LacZ and appears purple. Cell phone camera was used to capture the image of the multi-well plate at the end of 2h at 37°C. Absorbance at 576 nm is reported in Fig 3. IVTT with no sensor or trigger (Blank) and with a previously reported SARS-COV2 toehold sensor without (CVD sensor17- OFF) and with its cognate trigger (CVD sensor 17-ON) are used as controls. B. Additional replicates 2, 3 or 4 for time-course curves of sensors 3, 6 and 7 plotted in Fig 3C are shown here.
(EPS)

S4 Fig IVTT assay performed with sensors 3, 6 and 7 in the absence (black curve) or presence of various triggers from the trigger pool (colored lines, colors are matched with the left panel). IVTT was monitored through change in absorbance at 576 nm with 10^13^ copies of each trigger. Values above 4 have been plotted but should be looked at only for the overall trend and not for their absolute values. N = 2 replicate of the time-course profile are shown here (N = 1 shown in Fig 4A–4C).
(EPS)

S5 Fig Pie-chart illustrating the distribution of DENV serotypes detected (shades of green) by our pan-dengue sensors 3 (A), 6 (B), and 7 (C). Slice of the pie-chart highlights genomes in which no triggers were identified (black), genomes whose Triggers were not tested (red), and genomes that were tested but not detected by the sensor (gray).
(EPS)

S6 Fig Change in absorbance over time observed in IVTT assays performed with sensor 3 (A) and 6 (B) with varying amounts of their cognate trigger RNA (ranging from 10^13^ copies to 10^4^ copies of RNA). OFF state with no trigger is shown as a black dashed line. N = 2 replicate are shown.
(EPS)

S7 Fig NASBA reactions were performed on templates representing different serotypes- DENV Serotype 1 (A), DENV Serotype 2 (B), DENV Serotype 3 (C), and DENV Serotype 4 (D). NASBA reactions post 2 hours were added to an IVTT reaction containing sensor 3. Change in absorbance at 576 nm was recorded over time. NASBA reactions performed on the different templates (solid lines) were compared with reactions where no template was added to the NASBA (dashed lines). N = 2, 3 (or 4 where applicable). N = 1 is shown in Fig 5A–5D.
(EPS)

S8 Fig Change in absorbance over time observed in IVTT assays performed with sensor 3 and different RNAs including DENV Trg 3 RNA (red curve), human RNA (blue curve), COVID-19 RNA (orange curve), or without any RNA (black dashed curve). (EPS)

S1 Table Distribution of representative triggers A&B (Fig 2A, left), and trigger pools A&B (Fig 2A, right) across all serotypes of the DENV virus. (XLSX)

S2 Table Sequences of the and primers used in the construction of toehold sensors and corresponding triggers. (XLSX)

S3 Table Sequences and primers used to construct sequences tested in Fig 4A–4C. (XLSX)

S4 Table Primers used in NASBA and the sequence of templates used in the reaction. (XLSX)

S5 Table Sequences of primers used in this study. (XLSX)
